# Combined Poziotinib with Manidipine Treatment Suppresses Ovarian Cancer Stem-Cell Proliferation and Stemness

**DOI:** 10.3390/ijms21197379

**Published:** 2020-10-06

**Authors:** Heejin Lee, Jun Woo Kim, Dong-Seok Lee, Sang-Hyun Min

**Affiliations:** 1 New Drug Development Center, Daegu Gyeongbuk Medical Innovation Foundation (DGMIF), 80 Chumbok-ro, Dong-gu, Daegu 41061, Korea; free7e77@knu.ac.kr (H.L.); jwkim@dgmif.re.kr (J.W.K.); 2 BK21 Plus KNU Creative BioResearch Group, School of Life Sciences and Biotechnology, Kyungpook National University, Daegu 41566, Korea; lee1@knu.ac.kr

**Keywords:** drug synergism, poziotinib, manidipine, cancer stem cells, stemness, Wnt/β-catenin, STAT5, calcium channel blocker

## Abstract

Epithelial ovarian cancer (EOC) is the most lethal gynecological malignancy in women worldwide, with an overall 5 year survival rate below 30%. The low survival rate is associated with the persistence of cancer stem cells (CSCs) after chemotherapy. Therefore, CSC-targeting strategies are required for successful EOC treatment. Pan-human epidermal growth factor receptor 4 (HER4) and L-type calcium channels are highly expressed in ovarian CSCs, and treatment with the pan-HER inhibitor poziotinib or calcium channel blockers (CCBs) selectively inhibits the growth of ovarian CSCs via distinct molecular mechanisms. In this study, we tested the hypothesis that combination treatment with poziotinib and CCBs can synergistically inhibit the growth of ovarian CSCs. Combined treatment with poziotinib and manidipine (an L-type CCB) synergistically suppressed ovarian CSC sphere formation and viability compared with either drug alone. Moreover, combination treatment synergistically reduced the expression of stemness markers, including CD133, KLF4, and NANOG, and stemness-related signaling molecules, such as phospho-STAT5, phospho-AKT, phospho-ERK, and Wnt/β-catenin. Moreover, poziotinib with manidipine dramatically induced apoptosis in ovarian CSCs. Our results suggest that the combinatorial use of poziotinib with a CCB can effectively inhibit ovarian CSC survival and function.

## 1. Introduction

Epithelial ovarian cancer (EOC) is the fifth leading cause of cancer-related deaths among women worldwide, and it has the highest mortality rate of all gynecological malignancies [1,2]. Ovarian cancer therapy is more effective than therapies for other cancers, and it usually comprises surgery, chemotherapy, and sometimes radiotherapy [3,4]. Chemotherapy for ovarian cancer typically consists of platinum-based and taxane-based drugs [5,6]. In addition, PARP inhibitors, such as olaparib and niraparib, are used as targeted therapies [7,8]. Despite these treatment options, 50–70% of patients experience recurrence after treatment [9]. The recurrence may be explained by cancer stem cell (CSC) theory [10]. CSCs are cancer cells with the features of normal stem cells; they are responsible for the initiation, recurrence, and metastasis of cancer [11]. Moreover, CSCs possess self-renewal ability and are resistant to chemotherapy and radiotherapy [12,13,14]. Therefore, the elimination of ovarian CSCs is important for the prevention of ovarian cancer recurrence and prolongation of the survival of patients with ovarian cancer.

In previous studies, we used sphere-forming cells derived from A2780 and SKOV3 epithelial ovarian cancer cells (A2780-SP and SKOV3-SP cells), which have the characteristics of CSCs, to screen for drugs that target ovarian CSCs. Our screen identified four voltage-gated calcium channel blockers (CCBs; manidipine, lacidipine, lomerizine HCl, and benidipine HCl) and a pan-epithermal growth factor receptor (EGFR) inhibitor (poziotinib) that target ovarian CSCs [15,16]. The CCBs are approved by the United States (US) Food and Drug Administration as antihypertensive agents [17]. We confirmed that L/T-type calcium channels were overexpressed in ovarian CSCs, and that CCBs inhibited the sphere formation, viability, and proliferation of CSCs, as well as induced their apoptosis. In addition, CCBs suppressed stemness and inhibited the AKT and ERK signaling pathways in ovarian CSCs. Poziotinib is an inhibitor of epithermal growth factor receptors (EGFR/human EGFR 1 (HER1), 2, and 4) that is being developed by Hanmi Pharmaceutical; it is currently in global phase II clinical trials for the treatment of non-small-cell lung cancer [18,19]. We confirmed that HER4 was overexpressed in ovarian CSCs. Poziotinib blocked sphere formation, viability, and proliferation by ovarian CSCs, and induced G1 cell-cycle arrest and apoptosis. In addition, poziotinib suppressed the stemness of CSCs and disrupted the Wnt/β-catenin, Notch, and Hedgehog pathways, which contribute to the self-renewal of CSCs. However, combination treatment with poziotinib and cisplatin did not mediate synergistic effects on ovarian CSCs.

Drug combination therapies are widely used for the treatment of cancer. The main objectives of combination therapies are to achieve synergistic effects, reduce drug dose and toxicity, and minimize or delay the induction of drug resistance [20,21]. In this study, we used a combination of poziotinib and CCBs to target ovarian CSCs. We predicted that these drugs would have synergistic effects when combined, as they inhibit stemness through different signaling pathways. We analyzed the regulation of CSC sphere formation, viability, stemness, and apoptosis.

## 2. Results

### 2.1. Synergistic Effects of Poziotinib and CCBs on Ovarian Cancer and CSCs

The effects of poziotinib and manidipine on ovarian CSCs were tested in A2780-SP cells and SKOV3-SP cells, which were generated from A2780 and SKOV3 cells, respectively, cultured in low-attachment conditions in CSC medium. Compared with their parental EOC cell lines, A2780-SP and SKOV3-SP cells exhibited CSC characteristics. A2780-SP cells have higher messenger RNA (mRNA) and protein levels of stemness markers than A2780 cells [15,22], and SKOV3-SP cells expressed higher mRNA levels of stemness markers than SKOV3 cells (Figure S1A). In addition, A2780-SP cells expressed higher mRNA levels of *ERBB4* (HER4) [16], and SKOV3-SP cells expressed higher mRNA levels of *ERBB2* (HER2) and *ERBB4* (HER4) than their parent cells (Figure S1B).

**Figure 1:**
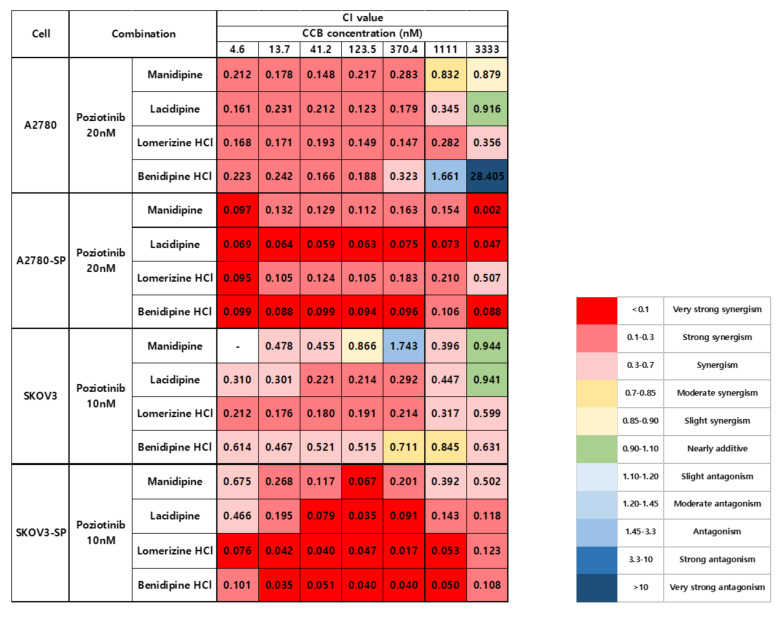
Combination index (CI) values for combination treatment with poziotinib and CCBs in ovarian cancer cells and ovarian CSCs.

**Figure 2:**
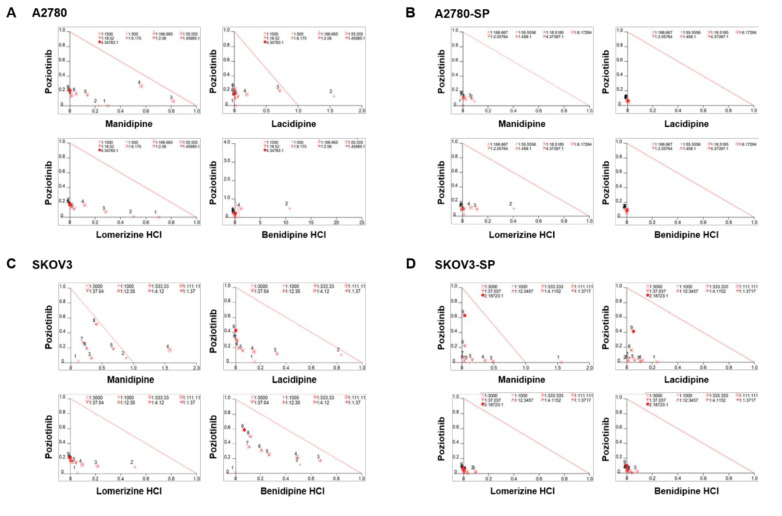
Isobolograms of poziotinib and calcium channel blocker (CCB) combination therapy for ovarian cancer cells and ovarian cancer stem cells (CSCs). (**A**–**D**) Normalized isobolograms of combination therapy with poziotinib and CCBs in A2780 cells (**A**), A2780-SP cells (**B**), SKOV3 cells (**C**), and SKOV3-SP cells (**D**). The data were analyzed using CalcuSyn software. The solid line represents additivity. Data points below the line indicate a synergistic interaction, and data points above the line indicate an antagonistic interaction. The numbers under the isobolograms indicate the concentrations of the drugs.

We treated the ovarian cancer cell lines and CSCs with poziotinib and CCBs, alone or in combination, for 6 days, and then determined the percentages of viable cells using an ATP-based assay (Figure S2). After calculating the concentrations of poziotinib and CCBs that caused 50% cell growth inhibition, we selected 10 nM (SKOV3 and SKOV3-SP cells) and 20 nM (A2780 and A2780-SP cells) poziotinib to reduce the viability of ovarian CSCs by 10–20% as a single agent, and a range of CCB concentrations (0–30 μM), for use in further experiments. The combination index (CI) values are shown in Figure 1, and the normalized isobolograms are shown in (Figure 2A–D). The CI values below the line represent synergy, whereas those close to the line represent additive effects, and those above the line represent antagonism. The combination therapy showed strong synergistic effects at all concentrations of CCBs in A2780-SP and SKOV3-SP cells (Figure 1; Figure 2B,D). We also observed synergistic effects in the parental A2780 and SKOV3 cells, but the effects were weaker for the higher concentrations of CCBs (Figure 1; Figure 2A,C). These findings suggest that the combination treatment strategy with poziotinib and CCBs is required to augment antitumor efficacy in the ovarian CSCs.

### 2.2. Combination Treatment with Poziotinib and Manidipine Synergistically Suppressed the Growth and Stemness of Ovarian CSCs

**Figure 3:**
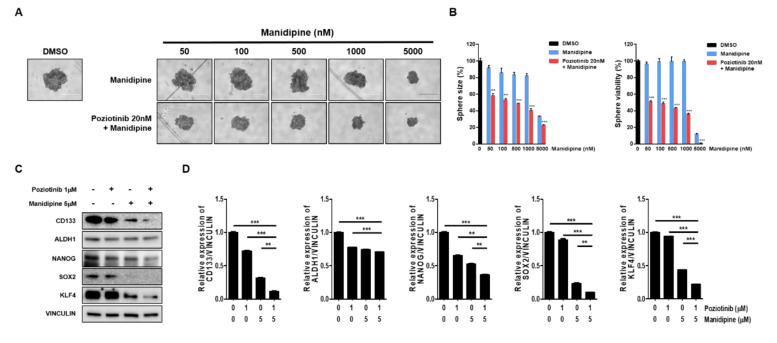
The effects of poziotinib and manidipine, alone or in combination, on ovarian CSC growth and expression of stemness markers. (**A**) A2780-SP cells were treated with poziotinib and manidipine at the indicated concentrations for 6 days. Representative image of spheres after 6 days of treatment. (**B**) Sphere size (left panel) quantitation and viability (right panel). The data are expressed as the mean ± SD of two or three independent experiments. ** *p* < 0.01, *** *p* < 0.001. (**C**) Protein expression levels of stemness markers were detected by Western blotting. A2780-SP cells were treated with poziotinib and manidipine at the indicated concentrations for 24 h. (**D**) The results of the Western blot are shown in a graph. The data are expressed as the mean ± SD of three independent experiments. ** *p* < 0.01, *** *p* < 0.001.

We next tested the effects of the combination treatment on the spheroid growth of ovarian CSCs. A2780-SP cells were seeded to form a sphere, and then treated with manidipine at the indicated concentration, alone or in combination with 20 nM poziotinib. After 6 days of treatment, sphere size and viability were measured. We found that manidipine dose-dependently decreased the sphere size and viability of A2780-SP cells. Moreover, combination treatment with poziotinib more effectively inhibited sphere size and viability than manidipine alone (Figure 3A,B). Next, we examined the effects of poziotinib and manidipine on the protein expression levels of stemness markers in ovarian CSCs. The combination treatment suppressed the expression of CD133, NANOG, SOX2, and KLF4 to a greater extent than treated alone (Figure 3C,D).

### 2.3. Combination Treatment Inhibited the Expression of Signaling Proteins Involved in Ovarian CSC Stemness

**Figure 4:**
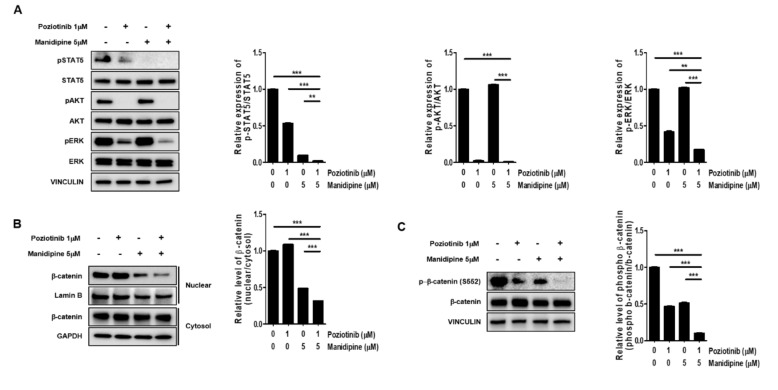
The effects of poziotinib and manidipine alone or in combination on the expression of stemness-related signaling molecules in ovarian CSCs. (**A**) Expression levels of phosphorylated proteins were detected by Western blotting. A2780-SP cells were treated with poziotinib and manidipine at the indicated concentrations for 24 h, and the results are shown in a graph (right panel). (**B**) Protein levels of cytosolic and nuclear β-catenin were detected in A2780-SP cells treated with poziotinib and manidipine by Western blotting. The results are shown in a graph (right panel). (**C**) Levels of phospho-β-catenin (S552) and total β-catenin were detected in A2780-SP cells treated with poziotinib and manidipine by Western blotting. The results are shown in a graph (right panel). The data are expressed as the mean ± SD of two or three independent experiments. ** *p* < 0.01, *** *p* < 0.001.

Next, we tested whether the combination treatment with poziotinib and manidipine affected the expression of stemness-related signaling proteins in ovarian CSCs. We measured the protein phosphorylation levels of STAT5, AKT, and ERK in ovarian CSCs after treatment for 24 h. Poziotinib combined with manidipine exerted a stronger inhibitory effect on protein expression than either single agent (Figure 4A). We also assessed the translocation of β-catenin after treatment with poziotinib or manidipine. The translocation of β-catenin to the nucleus in A2780-SP cells was inhibited by combination treatment compared with control or single-agent treatment (Figure 4B). In addition, concomitantly less phosphorylation of β-catenin on Ser 552, which is associated with decreased β-catenin translocation, was detected in A2780-SP cells after treatment with poziotinib or manidipine (Figure 4C). These results suggest that combination treatment of poziotinib and mandipine significantly downregulated the stemness-related signaling pathways of A2780-SP cells through simultaneous inhibition of the ErbB4 and L-type calcium channel.

### 2.4. Poziotinib Synergizes with Manidipine to Induce Apoptosis of Ovarian CSCs

**Figure 5:**
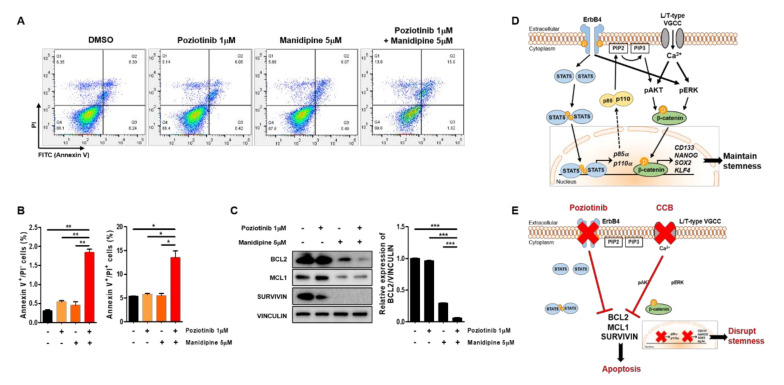
Apoptosis of ovarian CSCs induced by poziotinib and manidipine alone or in combination. (**A**) Apoptosis was analyzed by annexin V (AV)/propidium iodide (PI) staining in A2780-SP cells 24 h after treatment with the indicated concentrations of drugs. (**B**) The percentages of AV^+^/PI^−^ and AV^+^/PI^+^ cells are expressed as the mean ± SD of two or three independent experiments. * *p* < 0.05, ** *p* < 0.01, *** *p* < 0.001. (**C**) The expression of proapoptotic proteins was detected in A2780-SP cells treated with poziotinib and manidipine by Western blotting. The results of BCL2 expression are shown in a graph (right panel). The data are expressed as the mean ± SD of three independent experiments. * *p* < 0.05, ** *p* < 0.01, *** *p* < 0.001. (**D**) Schematic representation of the mechanisms of stemness maintenance in ovarian CSCs. (**E**) Schematic representation of the mechanisms of poziotinib and manidipine action in ovarian CSCs.

To determine whether treatment with poziotinib and manidipine, alone or in combination, affected the apoptosis of A2780-SP cells, we detected the percentages of cells in early apoptosis (annexin V (AV)^+^ and propidium iodide (PI)^−^) and late apoptosis (AV^+^/PI^+^) by flow cytometry. Treatment with poziotinib or manidipine alone induced low levels of apoptosis of A2780-SP cells (Figure 5A,B). However, poziotinib combined with manidipine significantly enhanced the induction of A2780-SP cell apoptosis. Furthermore, we confirmed that the expression of the antiapoptotic proteins BCL2, MCL1, and Survivin was lower in A2780-SP cells after combination treatment than in the control or poziotinib single-agent treatment groups (Figure 5C). In particular, the expression of BCL2 was dramatically reduced in A2780-SP cells after combination treatment than in the control or single-agent treatment groups (Figure 5C, right panel).

## 3. Discussion

Chemotherapy is one of the main comprehensive treatments used to slow tumor development and prolong the survival rate after surgery [23,24,25]. Usually, ovarian cancer patients can respond to a combination of taxane- and platinum-based chemotherapy when the disease is advanced, but chemo-resistant cancer cells can persist in metastatic sites, where they remain dormant for prolonged periods after initial therapy, eventually leading to relapse [26]. Despite ongoing efforts in developing surgery and intensive combination chemotherapy, long-term clinical outcomes in patients with advanced ovarian cancer have not significantly improved over the recent decades [27,28,29]. Clinically, ovarian cancer stem cells have been shown to survive in traditional chemotherapy [29]. In this regard, even though ovarian CSCs have not been completely elucidated, a small population of cancer cells with chemoresistance may possess cancer stem-cell properties and play a crucial role in recurrence [30,31]. Therefore, a novel strategy is needed to disrupt the cancer stem-cell properties and stem-cell signaling pathways.

In this study, we demonstrated that the combination of poziotinib and manidipine has synergistic effects on ovarian CSCs. We used the Chou–Talalay method to verify the synergistic effects of the combined treatment regimen and to select a reasonable dose. Synergism was observed more strongly in ovarian CSCs than in ovarian cancer cells for most concentrations of poziotinib and the CCBs. Combined treatment with poziotinib and manidipine inhibited the expression of stem-cell markers, especially CD133, NANOG, and KLF4. The two drugs also suppressed the phosphorylation of STAT5, AKT, and ERK, which are involved in the self-renewal ability of CSCs, as well as the translocation of β-catenin to the nucleus. In addition, combination treatment induced apoptosis in ovarian CSCs.

In ovarian CSCs, ErbB4 receptor tyrosine kinase and STAT5 cooperate to promote the transcription of p85a and p110a, which are subunits of PI3K [16]. AKT is phosphorylated downstream of PI3K signaling [32,33], and ERK phosphorylation is enhanced by overexpression of the ErbB4 receptor [16,34]. The overexpressed L/T-type calcium channels increase calcium flow into the cytoplasm, which drives phosphorylation of AKT and ERK through PI3K and MAPK signaling [15,35]. The phosphorylation of AKT and ERK stabilizes β-catenin, which translocates into the nucleus from the cytoplasm to increase transcription of CD133, NANOG, SOX2, and KLF4, thereby maintaining the stemness of ovarian CSCs (Figure 5D). Combination treatment with poziotinib and manidipine disrupts ovarian CSC stemness and induces apoptosis (Figure 5E). Our results demonstrate the interaction between the HER4 receptor and L/T-type calcium channels in maintaining ovarian CSCs.

In conclusion, our data suggest that combination treatment with poziotinib and manidipine synergistically inhibits ovarian CSCs through inhibition of HER4 and calcium channel-mediated STAT5, AKT, and ERK signaling. Although we plan to confirm the potential of this approach in vivo, our current findings highlight the promise of the proposed combination to counteract CSC-mediated chemoresistance.

## 4. Materials and Methods 

### 4.1. Two- and Three-Dimensional Spheroid Cell Cultures and Drug Treatment

The EOC cell lines A2780 and A2780-SP were supplied by Jae Ho Kim (Pusan National University, Korea). A2780 cells were cultured in RPMI-1640 medium supplemented with 10% fetal bovine serum (FBS) and 1% penicillin/streptomycin (all reagents from HyClone, Logan, Utah, USA). SKOV3 cells were purchased from the American Type Culture Collection (Manassas, VA, USA), and cultured in McCoy’s 5A medium (Gibco, Gaithersburg, USA) supplemented with 10% FBS (HyClone, Logan, Utah, USA) and 1% penicillin/streptomycin (HyClone, Logan, Utah, USA). A2780-SP and SKOV3-SP cells were cultured in complete medium (CM) comprising Neurobasal™ Medium (Gibco, Gaithersburg, USA) supplemented with B27 (Gibco, Gaithersburg, USA), 10 ng/mL basic fibroblast growth factor (R&D Systems, Minneapolis, MN, USA), 20 ng/mL human epidermal growth factor (R&D Systems, Minneapolis, MN, USA), GlutaMAX™ (Gibco, Gaithersburg, USA), HEPES (Gibco, Gaithersburg, USA), and 2.5 μg/mL amphotericin B (Gibco, Gaithersburg, USA) in ultra-low attachment (ULA) 100 mm^2^ plates (Corning, NY, USA). The sphere culture medium (CM) was changed every 2 to 3 days. Spheres were dissociated into single cells by treatment with Accutase^®^ (Gibco, Gaithersburg, USA). Poziotinib, manidipine, lacidipine, lomerizine HCl, benidipine HCl (MedChemExpress, Monmouth Junction, New Jersey, USA), and dimethyl sulfoxide (Sigma-Aldrich, St Louis, MO, USA) were used at the indicated concentrations.

### 4.2. Cell Viability Assay and CI Analysis

Ovarian cancer cells (A2780 and SKOV3) were seeded in white-bottom 96-well cell culture plates (Corning, USA) at a density of 3000 cells/well. After 24 h, the compounds were added to the cultures at the indicated concentrations. After 3 days, cell viability was assessed by CellTiter-Glo^®^ (Promega, Madison, WA, USA), and luciferase activity was detected using a Tecan plate reader (Biocompare, San Francisco, CA, USA).

A2780-SP and SKOV3-SP were seeded in ULA round-bottom 96-well plates (Corning, USA) at a density of 1800 cells/well. Then, the samples were centrifuged at 3000 rpm for 3 min. After 24 h, the compounds were added at the indicated concentrations. After 3 days, the cells were suspended in fresh medium containing the same concentrations of the compounds. Three days later, the sphere cells were imaged using an EVOS cell imaging system (Thermo Fisher Scientific, USA). We measured the sphere size using ImageJ software (NIH Image, USA). After imaging, sphere cell viability was assessed using CellTiter-Glo^®^ (Promega, Madison, WA, USA), and luciferase activity was detected using a Tecan plate reader (Biocompare, San Francisco, CA, USA).

The CI was calculated according to the method described by Chou and Talalay using CompuSyn software (COMBOSYN, Paramus, NJ, USA). First, we estimated the intercept and the slope from a dose–response curve and calculated the median effective dose (ED50). The ED50 was used to evaluate the CI, an essential measure for the determination of combination effects. Subsequently, we employed fraction-affected CI plots and isobologram analysis to determine whether the interactions between two compounds were additive, synergistic, or antagonistic. The fraction-affected CI plot and isobologram yield identical conclusions about synergism or antagonism. CI < 1 indicates synergism, CI = 1 indicates additive effects, and CI > 1 indicates antagonistic effects [36,37].

### 4.3. Western Blotting Assay

Protein extraction solution (radioimmunoprecipitation assay buffer with a phosphatase inhibitor and protease inhibitor cocktail) was used to obtain whole-cell lysates. Nuclear and cytosolic fractions were prepared using a Nuclear and Cytoplasmic Isolation kit (Thermo Fisher Scientific, San Jose, CA, USA). Cell lysates were separated using sodium dodecyl sulfate polyacrylamide gel electrophoresis and transferred to polyvinylidene fluoride membranes for Western blot analysis. After blocking with 5% skim milk, the membranes were incubated with primary antibodies in blocking buffer overnight at 4 °C, followed by incubation with horseradish peroxidase (HRP)-conjugated secondary antibodies for 2 h at room temperature. The following primary antibodies against the specified antigens were used: CD133 (Abcam, Cambridge, MA, USA, ab19898), ALDH (Santa Cruz Biotechnology, CA, USA, sc-166362), NANOG (Cell Signaling Technology, Danvers, MA, USA, #3580S), SOX2 (Cell Signaling Technology, Danvers, MA, USA, #2748S), KLF4 (GeneTex, Texas, USA, GTX101508), vinculin (Santa Cruz Biotechnology, CA, USA, sc-73614), phospho-STAT5 (Y694) (Cell Signaling Technology, Danvers, MA, USA, #9351S), STAT5 (Cell Signaling Technology, Danvers, MA, USA, #94205S), phospho-AKT (Ser473) (Cell Signaling Technology, Danvers, MA, USA, #3787S), AKT (Cell Signaling Technology, Danvers, MA, USA, #9272S), phospho-ERK (Thr202/Tyr204) (Cell Signaling Technology, Danvers, MA, USA, #9101S), ERK (Cell Signaling Technology, Danvers, MA, USA, #9102S), β-catenin (Cell Signaling Technology, Danvers, MA, USA, #8480S), lamin B (Abbkine, California, USA, #A01090), GAPDH (Santa Cruz Biotechnology, CA, USA, sc-47724), phospho-β-catenin (S552) (Cell Signaling Technology, Danvers, MA, USA, #9566S), BCL2 (Santa Cruz Biotechnology, CA, USA, sc-7382), MCL1 (Cell Signaling Technology, Danvers, MA, USA, #4572S), and Survivin (Cell Signaling Technology, Danvers, MA, USA, #2803S). The secondary antibodies used were goat anti-mouse IgG-HRP (R&D Systems, Minneapolis, MN, USA) and goat anti-rabbit IgG-HRP (R&D Systems, Minneapolis, Minnesotta, USA). Signals were developed with enhanced chemiluminescence HRP substrate (Bio-Rad Laboratories, Hercules, CA, USA) and detected using a LAS-3000 Mini (Fujifilm, Tokyo, Japan). The signal intensities were calculated using ImageJ software (NIH Image, Wayne Rasband, NIH, Bethesda, MD, USA).

### 4.4. Analysis of Apoptosis (AV/PI Staining) 

A2780-SP cells were seeded in ULA six-well plates at a density of 1 × 10^6^ cells/well and grown in CSC medium. After 24 h, the A2780-SP cells were treated with the compounds. The sphere cultures were grown for 24 h at 37 °C in 5% CO_2_ and 95% humidity. After 24 h, the cells were harvested by centrifugation. Spheres were dissociated into single-cell suspensions by treatment with Accutase^®^. The supernatants were decanted, and then the cells were gently resuspended and washed once in phosphate-buffered saline. The cell pellets were resuspended in 0.1 mL of annexin V binding buffer (BD Biosciences, San Diego, CA, USA). We added 5 μL of FITC annexin V and 5 μL of PI (BD Biosciences, San Diego, CA, USA), and gently vortexed the cells. The cells were incubated for 15 min in the dark at room temperature, and then 400 μL of annexin V binding buffer was added to each tube. Flow cytometry was performed within 1 h.

### 4.5. RNA Isolation and Quantitative Reverse Transcription PCR

Total RNA was extracted from the samples using a TRIzol™ RNA extraction kit (GeneAll, USA) according to the manufacturer’s instructions. We reverse-transcribed 1 μg of the RNA into complementary DNA (cDNA) using a cDNA reverse transcription kit (Applied Biosystems, Forster City, CA, USA). The synthesized cDNA was amplified by quantitative real-time polymerase chain reaction (PCR) using SYBR™ Green PCR Master Mix and a StepOne™ Real-Time PCR system (Applied Biosystems, Forster City, CA, USA) with the indicated primers. *GAPDH* was used as the reference gene. The results are presented relative to the control using the 2^−ΔΔCt^ method. The primers used in these experiments were *GAPDH* (forward (F): 5′–GGAGCCAAAAGGGTCATCAT–3′, reverse (R): 5′–GTGATGGCATGGACTGTGGT–3′), *CD133* (F: 5′–AGTCGGAAACTGGCAGATAGC–3′, R: 5′–GGTAGTGTTGTACTGGGCCAAT–3′), *ALDH1* (F: 5′–CTCGAAATTAAGTACACCAA–3′, R: 5′–TCAGTAGACCCTGTGAATGC–3′), *OCT3/4* (F: 5′–GACAACAATGAAAATCTTCAGGAGA–3′, R: 5′–TTCTGGCGCCGGTTACAGAACCA–3′), *NANOG* (F: 5′–TGCCTCACACGGAGACTGTC–3′, R: 5′–TGCTATTCTTCGGCCAGTTG–3′), *SOX2* (F: 5′–GGGAAATGGGAGGGGTGCAAAAGAGG–3′, R: 5′–TTGCGTGAGTGTGGATGGGGATTGGTG–3′), *KLF4* (F: 5′–CCTACCTCGGAGAGAGACCG–3′, R: 5′–GGACTCCCTGCCATAGAGGA–3′), *EGFR* (F: 5′–AGGCACGAGTAACAAGCTCAC–3′, R: 5′–ATGAGGACATAACCAGCCACC–3′), *ERBB2* (F: 5′–TGTGACTGCCTGTCCCTACAA–3′, R: 5′–CCAGACCATAGCACACTCGG–3′), and *ERBB4* (F: 5′–GCAGATGCTACGGACCTTACG–3′, R: 5′–GACACTGAGTAACACATGCTCC–3′).

### 4.6. Statistical Analysis

All data are expressed as the mean ± standard deviation of ≥2 independent experiments. Statistically significant differences were determined using a *t*-test or one-way analysis of variance with GraphPad Prism 5 (GraphPad, La Jolla, CA, USA). A difference with a *p*-value <0.05 was considered statistically significant.

## Supplementary Materials

The following are available online at https://www.mdpi.com/1422-0067/21/19/7379/s1: Figure S1. Expression of stemnessmarkers and EGFR family mRNA levels in SKOV3 and SKOV3-SP cells. Figure S2. Growth inhibition of poziotiniband manidipinein ovarian cancer cells and CSCs.

## Abbreviations

EOA	Epithelial ovarian cancer
CSC	Cancer stem cell
CCB	Calcium channel blocker
HER4	Human epithermal growth factor receptor 4
EGFR	Epidermal growth factor receptor
CI	Combination index
AV	Annexin V
PI	Propidium iodide
CM	Complete medium
ULA	Ultra-low attachment
ED50	Median effective dose
HRP	Horseradish peroxidase
PCR	Polymerase chain reaction
F	Forward
R	Reverse
