# Supplementary material for: Combined Poziotinib with Manidipine Treatment Suppresses Ovarian Cancer Stem-Cell Proliferation and Stemness
Source: Int J Mol Sci. 2020 Oct 6;21(19):7379. doi: 10.3390/ijms21197379 (PMC7583017; doi:10.3390/ijms21197379)
Supplement: Supplementary file 1 [file ijms-21-07379-s001.pdf]

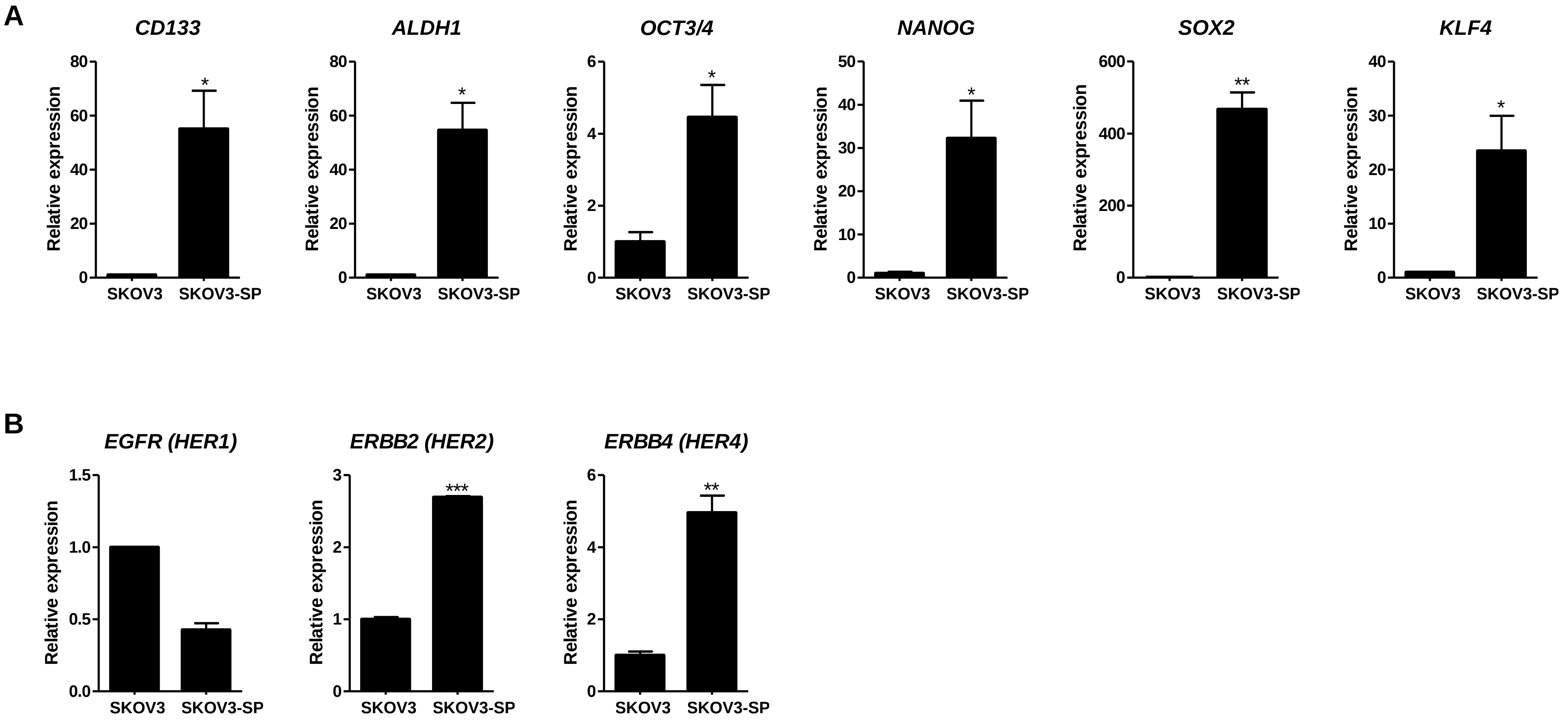

Supplementary Figure S1. Expression of stemness markers and EGFR family mRNA levels in SKOV3 and SKOV3-SP cells.

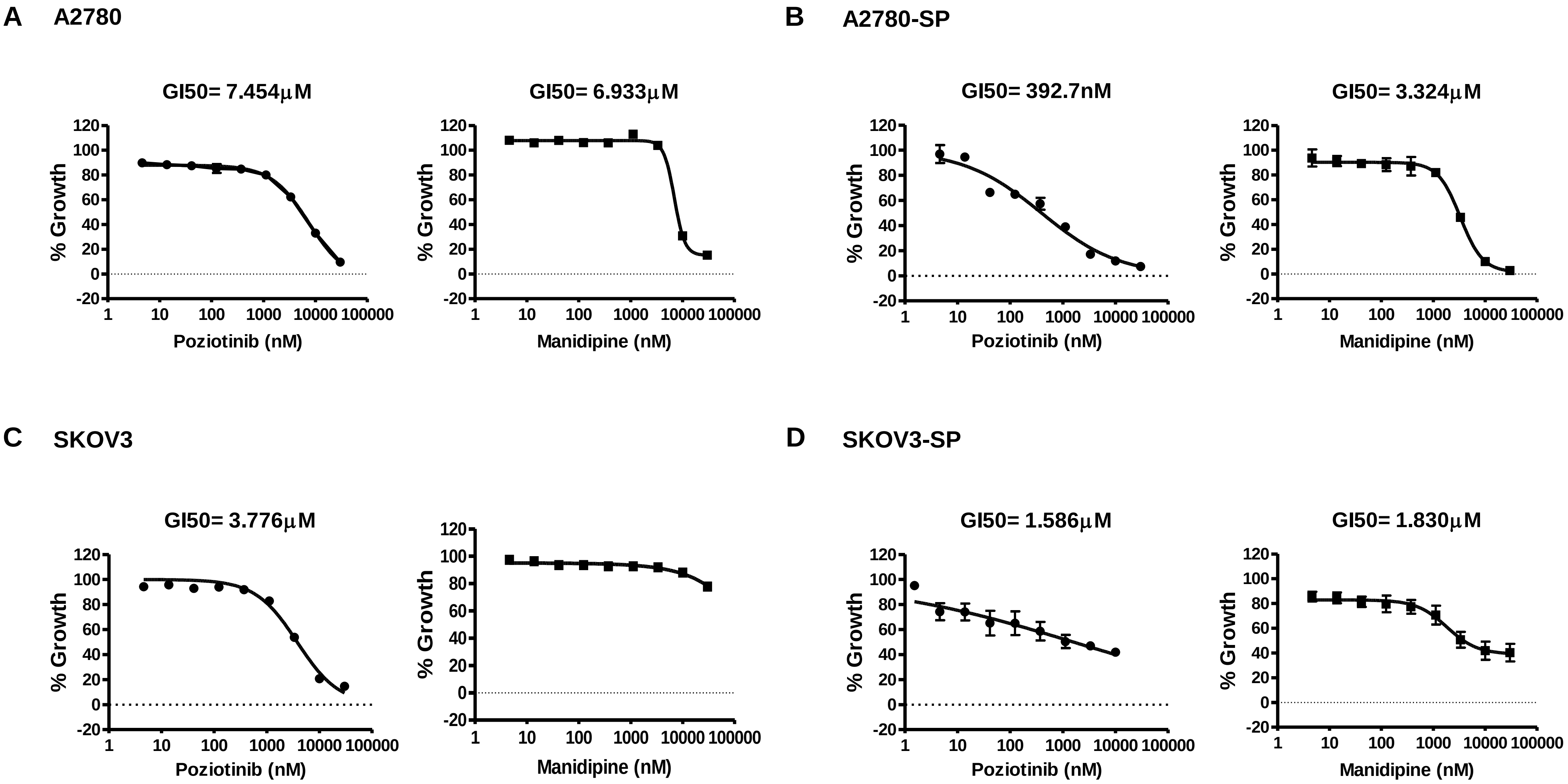

Supplementary Figure S2. Growth inhibition of poziotinib and manidipine in ovarian cancer cells and CSCs.
